# Electrospun Multilayered Films Based on Poly(3-hydroxybutyrate-*co*-3-hydroxyvalerate), Copolyamide 1010/1014, and Electrosprayed Nanostructured Silica

**DOI:** 10.3390/nano13060972

**Published:** 2023-03-08

**Authors:** Chiara Marcoaldi, Maria Pardo-Figuerez, Cristina Prieto, Carmen Arnal, Sergio Torres-Giner, Luis Cabedo, Jose M. Lagaron

**Affiliations:** 1 Novel Materials and Nanotechnology Group, Institute of Agrochemistry and Food Technology (IATA), Spanish Council for Scientific Research (CSIC), Calle Catedrático Agustín Escardino Benlloch 7, 46980 Paterna, Spain; 2 Polymers and Advanced Materials Group (PIMA), Universitat Jaume I (UJI), Avenida de Vicent Sos Baynat s/n, 12071 Castellón, Spain

**Keywords:** PHBV, electrospinning, biopolyamides, nanosilica, food packaging

## Abstract

In this research, bio-based electrospun multilayered films for food packaging applications with good barrier properties and close to superhydrophobic behavior were developed. For this purpose, two different biopolymers, a low-melting point and fully bio-based synthetic aliphatic copolyamide 1010/1014 (PA1010/1014) and the microbially synthesized poly(3-hydroxybutyrate-*co*-3-hydroxyvalerate) (PHBV) and food-contact-complying organomodified silica (SiO_2_) nanostructured microparticles, were processed by electrospinning. The production of the multilayer structure was finally obtained by means of a thermal post-treatment, with the aim to laminate all of the components by virtue of the so-called interfiber coalescence process. The so developed fully electrospun films were characterized according to their morphology, their permeance to water vapor and oxygen, the mechanical properties, and their water contact angle properties. Interestingly, the annealed electrospun copolyamide did not show the expected improved barrier behavior as a monolayer. However, when it was built into a multilayer form, the whole assembly exhibited a good barrier, an improved mechanical performance compared to pure PHBV, an apparent water contact angle of ca. 146°, and a sliding angle of 8°. Consequently, these new biopolymer-based multilayer films could be a bio-based alternative to be potentially considered in more environmentally friendly food packaging strategies.

## 1. Introduction

Plastics are widely recognized as having an ever-increasing weight in waste management. For this reason, several initiatives have been stimulated to tackle these problems, especially for plastic packaging, since it makes up the largest share of the post-consumer plastic waste stream [1]. To overcome these issues, biopolymers have been proposed to substitute plastics in eco-friendly packaging. They have been demonstrated to possess the adequate properties to provide physical protection, creating proper physico-chemical conditions to extend food shelf life, therefore reducing food loss and, at the same time, environmental pollution [2]. Moreover, biopolymers are in line with circular economy strategies, since they can be obtained through the reuse of waste as a raw material and the implementation of low-energy-consumption processes to produce recyclable and compostable packaging materials.

Additionally, the new generation of smart food packaging based on biopolymers is achieving great interest due to environmental pollution, and also to the large amount of food waste that is produced annually. In this regard, researchers are now focused on the development of innovative biodegradable packaging for application in the food industry to develop more effective food packaging items in order to extend food shelf life and reduce food and plastic waste [3]. Recent research has shown that this new generation of packaging is frequently composed of multilayer films with an improved functional performance with respect to monolayer materials. This type of packaging material usually consists of at least three distinct layers, i.e., the barrier, the active, and the structural layers [4]. These peculiar structures, with passive and active properties, can be made by combining biopolymers in order to achieve the desired properties without using fossil-derived materials. Furthermore, in the development of this new generation of food packaging material, another interesting characteristic is providing the film with hydrophobic and oleophobic properties. This represents an important feature in food packaging, since it allows the control of water loss in fruits and vegetables and prevents moist migration, and even flavor losses, as well as a better use of the packaged product. From the technological point of view, it is possible to obtain this kind of surface by altering the chemistry of the surface to achieve hydrophobic properties or by producing nano- or micro-roughened surfaces by the use of several techniques. 

In this sense, electrohydrodynamic processing (EHDP), such as electrospinning and electrospraying technologies, has proven to be a simple and versatile technique for manufacturing novel materials for different applications [5,6,7], among which the development of smart food packaging materials with enhanced functional properties stands out. By means of this technique, it is possible to produce micro- and/or nanostructures from a wide range of polymers in a versatile and controlled manner [8]. In this sense, the use of EHDP represents a way to overcome the disadvantages of the majority of the methods that are commonly used to obtain smart food packaging materials with enhanced functional properties, as proven in previous research. For example, Cherpinski et al. [9] developed oxygen-scavenging multilayered biopapers containing palladium nanoparticles (PdNPs), which were obtained by the electrospinning coating technique. In another study, Meléndez-Rodríguez et al. [10] studied the production of high-oxygen-barrier multilayer films based on polyhydroxyalkanoates (PHAs) and cellulose nanocrystals (CNCs). Furthermore, Figueroa et al. [11] developed active barrier multilayer films that were based on antimicrobial hot-tack electrospun PHAs and CNC interlayers. Finally, Lafraya et al. [12] produced super-repellent paper that was coated with electrospun biopolymers and electrosprayed silica, or silicon dioxide (SiO_2_), microparticles of application in the development of novel food packaging materials.

The objective of this work was to produce bio-based electrospun multilayered films for the development of novel food packaging materials with good barrier and superhydrophobic properties. The novelty of the work lies in the fact that the intermediate layer was made of a fully bio-based copolyamide with a low melting point and an improved flexibility [13]. Thus, unlike other higher barrier polyamide grades, such as PA6, the synthesized copolyamide that was selected in this study aimed to offer a bio-based origin, some barrier improvement, and flexibility to the multilayer, while allowing lamination with biopolyesters such as poly(3-hydroxybutyrate-co-3-hydroxyvalerate) (PHBV), which has a comparable melting point [14,15]. PHAs, such as PHBV, are promising aliphatic polyesters, because of their renewability, biodegradability, and biocompatibility [16]. Regarding their superhydrophobic properties, as well known in the literature, most of the materials that are already employed to obtain them are not suitable for food packaging applications [12]. In this work, a superhydrophobic coating was produced with nanostructured SiO_2_ microparticles, which are biocompatible and nontoxic. The hydrophobic SiO_2_ particles were electrosprayed on top of the PHBV layer to finally obtain a hierarchical micro/nanostructured surface. The morphological, optical, barrier, superhydrophobic, thermal, and mechanical properties of the produced electrospun multilayered films were evaluated in order to ascertain their potential in food packaging.

## 2. Materials and Methods

### 2.1. Materials

Commercial PHBV (ENMATTM Y1000P) in pellet form was purchased from Tianan Biologic Materials (Ningbo, China). As reported by the manufacturer, this biopolymer is characterized by a density of 1.23 g/cm^3^, a melt flow index (MFI) of 5–10 g/10 min (190 °C, 2.16 kg), and a 3 HV fraction of 2 mol %. Decanedioic acid, commonly known as sebacic acid (SbA) (molecular weight (M_W_) of 202.25 g/mol and 99% purity), decamethylenediamine (DMDA) (M_W_ of 172.31 g/mol and purity of 97%), tetradecanedioic acid (TTDDA) (M_W_ of 258.35 g/mol and purity of 99%), and 4-dimethylaminopyridine (DMAP) (M_W_ of 122.17 g/mol and purity of ≥99%) were all purchased at Sigma-Aldrich S.A. (Madrid, Spain). Sulfuric acid (96%) was provided by Vidrafroc S.A. (Barcelona, Spain) Sipernat D17 nanostructured SiO_2_ microparticles, organomodified with dimethyldichlorosilane (≥97%), were provided by Evonik (Essen, Germany) and are claimed by the manufacturer to be of food-contact grade. The 1-butanol (BuOH) was acquired from Merck (Darmstadt, Germany). The 2,2,2-trifluoroethanol (TFE) (≥99%), methanol (MeOH) (≥99.8%), and formic acid (≥95%) were also obtained from Sigma-Aldrich S.A. (Madrid, Spain), whereas hexafluoro-2-propanol (HFIP) was obtained from Fluorochem (Hadfield, UK). Absolute ethanol and methanol (99.8%) were from Labkem-Labbox (Mataró, Spain). All of the reagents were used without further purification.

### 2.2. Synthesis of the Bio-Based Copolyamide

All of the monomers used are of natural origin, since SbA and DMDA are both derived from castor oil, whereas TTDDA is obtained from tetradecane, a natural product found in eukaryotes and other organisms. The production of the biopolyamide started with prepolymer synthesis, based on the precipitation of the nylon salt from the reaction of two diacids, SbA and TTDDA, with DMAP diamine. An equimolar ratio of 0.5 moles of each diacid to 1 mole of diamine was used. For that, a 13 wt.% solution of diacids in ethanol was first prepared under stirring conditions at 40–45 °C. Then, a 34 wt.% diamine solution in ethanol was prepared under stirring conditions at room temperature. Thereafter, once both solutions were completely dissolved, the DMAP catalyst was added to the diacid solution at 0.5 wt.%, with respect to the total mixture of reagents (diacids and diamine). Subsequently, the diamine solution was added dropwise to the previous diacid solution containing the catalyzer, maintaining stirring and controlling the temperature at 40–45 °C for 30 min, and then keeping the resultant mixture in an ice bath for 1 h to precipitate the nylon salt. The white precipitate was washed with 200 mL of EtOH under stirring conditions at room temperature for 2 h and was then filtered and washed until reaching pH 6–7. Finally, it was dried in a vacuum oven (Vaciotem-T, J.P. Selecta, Barcelona, Spain) at 40 °C for 10 h.

PA1010/1014 was then synthesized from the resultant salt by melt polycondensation. For that, 30 g of nylon salt with 0.2% DMAP catalyst were placed in a 100 mL thermostated glass reactor connected to a Huber Pilot ONE^®^ CC^®^-304B (Peter Huber Kältemaschinenbau AG, Offenburg, Germany), coupled with mechanical stirring (RZR1, Heidolph Instruments GmbH & Co. KG, Schwabach, Germany), a gas inlet, and a distillation system. The reactor was closed, and the internal atmosphere was purged with a stream of nitrogen at a flowrate of 50 mL/min, keeping the mixture of solids under stirring conditions for 60 min to achieve an inert atmosphere. The system was then heated from room temperature to 220 °C at 3 °C/min. Once a temperature of 220 °C was reached, the reaction proceeded for 4.5 h under a nitrogen atmosphere at a flowrate of 50 mL/h. After this reaction was completed, the reactor was allowed to cool down to room temperature under the nitrogen atmosphere. Finally, the sample was removed from the reactor and crushed in an IKA A11 Basic Analytical mill (IKA^®^-Werke GmbH & Co. KG, Staufen, Germany), using liquid nitrogen to control temperature raise. The obtained copolyamide powder was washed with a mixture of 1000 mL of distilled water and 500 mL of EtOH in a beaker at 40 °C under stirring conditions for 8 h. It was then filtered and washed with 50 mL of MeOH to remove the remaining DMAP. Finally, the clean powder was dried for 8 h at 60 °C in a vacuum oven (Vaciotem-T, J.P. Selecta, Barcelona, Spain).

### 2.3. Polyamide Characterization

#### 2.3.1. ATR–FTIR

Attenuated total reflection–Fourier transform infrared (ATR–FTIR) spectra were collected, coupling the ATR auxiliary Golden Gate of Specac, Ltd. (Orpington, UK) to the Tensor 37 FTIR device (Bruker, Ettlingen, Germany). The spectra were obtained from 4000 to 600 cm^−1^ by averaging 20 scans with a resolution of 4 cm^−1^. The software OPUS 4.0 (Bruker, Ettlingen, Germany) was employed to analyze the spectral data.

#### 2.3.2. DSC

The thermal analysis was performed by differential scanning calorimetry (DSC) by means of a DSC 8000 analyzer from PerkinElmer, Inc. (Waltham, MA, USA) coupled to a cooling accessory (Intracooler 2, PerkinElmer, Inc.). The heating ramp was set from 30 to 270 °C in a nitrogen atmosphere, at a heating rate of 10 °C /min. An empty aluminum cup was used as a reference. The calibration was carried out with indium. The analysis was performed at least in triplicate. The percentage of crystallinity (X_c_) has been calculated by following Equation (1):

X_c_ = (∆H experimental/∆H perfect crystal) × 100 (1)

where ∆H experimental is the melting peak enthalpy determined experimentally for the produced polyamide and ∆H perfect crystal is the melting enthalpy of the perfect crystal for the same polyamide degree. The ∆H perfect crystal for polyamide 1010 has been reported to be 244 J/g [17].

#### 2.3.3. Viscosity Number and Molecular Weight

The viscosity number (VN) of the synthetized bio-based copolyamide was determined according to ISO 307:2007 standard, using a Type II Ubbelohde viscometer (Vidrafoc S.A., Barcelona, Spain). For this, a given amount of the polymer was first dissolved in concentrated sulfuric acid (96%). The solution was then placed into the viscometer, kept immersed in a water bath at 25 °C, and the time taken for the solution to flow between the two graduated marks was measured. The test was performed in duplicate. The viscosity number was calculated using Equation (2).

(2) $$\mathrm{VN}=\left[\left(\frac{\eta }{\eta_{0}}\right)-1\right]\times \frac{1}{C}$$

where η (N/m^2^·s) and η_0_ (N/m^2^·s) are the viscosity of the polymer solution in the specified acid and the viscosity of the solvent, respectively, whereas η/η_0_ is the relative viscosity of the polymer solution and C (0.005 g/mL) is the concentration of the polymer solution.

The value of M_W_ (g/mol) was estimated using Equation (3), according to the Mark–Houwink–Sakurada expression, as follows:

(3) $$\eta =K\times M_{W}{}^{\alpha }$$

where η (cm^3^/g) is the intrinsic viscosity, or Staudinger index, and K and α are the coefficient and molecular parameters that describe the hydrodynamic interaction between the macromolecules and solvent, respectively. The M_W_ value was estimated using the previous experimental data reported for PA6 [18] and PA12 [19].

### 2.4. Fabrication of the Nanostructured Coatings

#### 2.4.1. Preparation of the Polymer Solutions

Before electrospinning, the different polymer solutions and particle dispersion were prepared. PHBV was dissolved at a concentration of 10 wt.% in TFE. Formic acid was initially used to try to prepare the PA1010/1014 solution, according to the literature based on polyamide 6 (PA6) [20]; however, it was not successful, due to the lower hydrophilicity of the synthesized copolyamide. Thus, HFIP was used, and the amount of copolyamide was tested from 6 wt.% to 15 wt.%. The copolyamide solutions were homogenized overnight at 20 °C, and the most optimal results were attained for a concentration of 6 wt.%, since higher concentrations were too viscous and lower concentrations led to instabilities during EHDP [19]. The silica dispersion, at a concentration of 3 wt.% SiO_2_ in BuOH, was sonicated at a power of 40% for 5 min in a Sonopuls HD 2200.2 (Scharlab S.A., Barcelona, Spain).

#### 2.4.2. Solution Characterization

The biopolymer solutions were characterized in terms of viscosity, surface tension, and conductivity. The viscosity was determined using a rotational viscosity meter known as Visco BasicPlus L from Fungilab S.A. (San Feliu de Llobregat, Spain). The surface tension was determined using the Wilhelmy plate method in an Easy Dyne K20 tensiometer (Krüss GmbH, Hamburg, Germany). Finally, the conductivity was measured by means of a conductivity meter Xs Con6 (Hanna Instruments, Woonsocket, RI, USA).

#### 2.4.3. Electrospinning and Electrospraying Parameters

**Figure 1:**
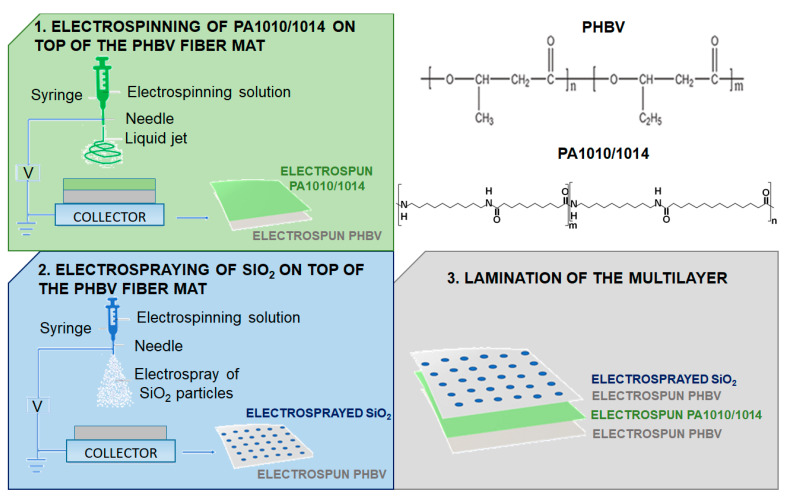
Scheme of the electrospun multilayered film production process and chemical structure of the biopolymers used.

The obtained solutions were processed in a Fluidnatek^TM^ LE-100 apparatus (Bioinicia S.L., Valencia, Spain) with a multi-emitter injector system, a drum collector, and with controlled temperature and relative humidity. Different processing conditions were tested in order to improve the fiber formation, for instance, the flow rate was studied between 1 and 5 mL/h, the voltage in the emitter between 5 and 20 kV, the voltage in the collector between 10 and 25 kV, and the distance between the emitter and the collector between 15 and 30 cm. See the selected conditions for each material in the results section below. The speed of the drum collector was maintained at 200 rpm, and the temperature and humidity at 30 °C and 30% RH, respectively. The PA1010/1014 fibers were deposited over a pre-electrospun mat of PHBV, and the silica was also electrosprayed on top of a pre-electrospun mat of PHBV. Figure 1 shows a scheme of the electrospun multilayered film production process.

#### 2.4.4. Thermal Post-Treatment

The resultant fiber mats were laminated by annealing by means of a thermal laminator known as Vansda® EL-520 (Jishi, China). Table 1 shows the conditions of the applied thermal treatment for both mono- and multilayer films. The optimum was considered as the lowest temperature below the polymer melting point that led to a continuous film by the so-called interfiber coalescence process. The PA1010/1014 fibers were dried at 90 °C under vacuum conditions overnight before lamination.

### 2.5. Coating Characterization

#### 2.5.1. Thickness

The thickness of all of the structures was measured prior to characterization, using a digital micrometer S00014 from Mitutoyo Corp. (Kawasaki, Japan), with an accuracy of ± 0.001 mm. The measurements were performed at five different points of the samples, including two measurements on each side and one measurement in the middle of the sample. The results shown are the average and standard deviation of these measurements.

#### 2.5.2. Morphology

The morphologies of the electrospun fibers and films were observed by scanning electron microscopy (SEM) by means of an S-4800 device (Hitachi, Tokyo, Japan). The samples were fixed to the holders using carbon double-sided adhesive tape and sputtered with a mixture of gold–palladium under vacuum conditions prior to observation, at an accelerating voltage of 10 kV. For the cross-section observations, the films were cryo-fractured in liquid nitrogen beforehand. The size distribution of the particles and average fiber diameter were determined via Image J Launcher v1.41 software (National Institutes of Health, Bethesda, MA, USA), using at least 20 SEM images.

#### 2.5.3. Optical Properties

The optical properties of the multilayers were determined in specimens of 50 mm × 30 mm by quantifying the absorption of light at wavelengths between 200 and 700 nm [21,22] in an ultraviolet–visible (UV–Vis) spectrophotometer VIS3000 from Dinko Instruments (Barcelona, Spain). The transparency (T) was evaluated using Equation (4) [22].

(4) $$T=\frac{A_{600}}{L}$$

where A_600_ is the absorbance value at 600 nm and L is the film thickness (mm).

An alternative method to assess the transparency (T) (see Equation (5)) was also used, which is based on an ASTM standard [21]. Although the ASTM standard recommends the use of absorbances in the range of 540–560 nm, we have used the value at 600 nm for comparison purposes, as follows:

(5) $$T=10^{\left(2-A_{600}\right)}$$

where A_600_ is the absorbance value at 600 nm.

#### 2.5.4. Water Contact Angle

The surfaces were classified as either hydrophobic and superhydrophobic according to the water contact angle (WCA or θ). Thus, a surface with θ > 90° was considered to be hydrophobic, while if the water contact angle was >150°, and the sliding angle (SA) was lower than 5° or 10°, it was considered superhydrophobic [23,24]. The apparent water contact angle was determined by means of a Video-Based Contact Angle Meter Theta Lite TL 101 model (Biolin Scientific, Espoo, Finland) and the images were analyzed by means of OneAttension software v 3.1 (Biolin Scientific, Espoo, Finland). A 5 µL drop of distilled water was deposited over the sample surface and data were recorded 20 s after the deposition of the droplet at room temperature. The measurements were carried out by averaging the measurements at 3 different positions.

#### 2.5.5. Sliding Angle

Sliding angle determination was performed at room temperature using a 15 µL water droplet that was deposited over the surface, which was recorded while being tilted. The images were analyzed with Image J v 1.52a software (National Institutes of Health, Bethesda, MA, USA).

#### 2.5.6. Water Vapor Permeance

Payne permeability cups (Elcometer SPRL, Lieja, Belgium) of 3.5 cm diameter were used to measure the water vapor permeance (WVP) of the samples, according to the ASTM gravimetric method (ASTM E96/E96M) [25] using Payne permeability cups at 100% relative humidity (RH). The Payne cups were stored in a desiccator at 25 °C and 0% RH, and their weight was measured periodically until a steady weight was reached. Aluminum films were used as a control to estimate the solvent loss through the sealing. The measurements were performed in triplicate. WVP was calculated from the steady-state slope obtained from the regression analysis of the weight loss data over time and corrected by leakage. For comparison purposes, a bilayer of PHBV/PHBV was used in the study.

#### 2.5.7. Oxygen Permeance

The oxygen permeance (OP) coefficient was derived from the oxygen transmission rate (OTR) measurements that were recorded using an Oxygen Permeation Analyzer M8001 from Systech Illinois (Thame, UK) at 60% RH and 25 °C, in duplicate, and according to the ASTM D3985-05 standard. The PA1010/1014 samples were also tested at 0% RH and 25 °C. The samples were purged with nitrogen before exposure to an oxygen flow of 10 mL/min. The exposure area during the test was 5 cm^2^ for each sample.

#### 2.5.8. Water Uptake

The water uptake of the multilayer structures was measured according to the standard method ASTM D570 [26]. The samples were first vacuum dried overnight at 100 °C and then placed in a desiccator at 0% RH for 24 h. Immediately after, the films were weighed and put into a desiccator at 100% RH and room temperature. The samples were weighed periodically after blotting the surface in an analytical balance until equilibrium was reached; this being determined when repeated measurements yielded the same weight. Equation (6) was used to measure the weight gain, as follows:

W (%) = (m_1_ − m_0_)/m_0_ × 100 (6)

where m_1_ is the sample weight at equilibrium and m_0_ is the initial sample weight.

#### 2.5.9. Mechanical Tests

Mechanical tests in dogbone-shaped specimens of the various samples cut in the machine direction (MD) and transversal direction (TD) were performed in a universal testing machine known as AGS-X 500N (Shimatzu, Kyoto, Japan) at room temperature with a cross-head speed of 10 mm/min, according to the ASTM D638 (Type IV) standard. Prior to the analysis, all of the samples were conditioned at room temperature and 0% RH. For comparison purposes, a bilayer of PHBV/PHBV was used in the study. At least six specimens per sample were tested, with the results being shown as the average and standard deviation of these six tests.

### 2.6. Statistical Analysis

The significance of the results was assessed via an analysis of variance (ANOVA) via Origin v9 software (OriginLab, Northampton, MA, USA), followed by a Tukey test (*p <* 0.05).

## 3. Results and Discussion

### 3.1. Polyamide Characterization

**Figure 2:**
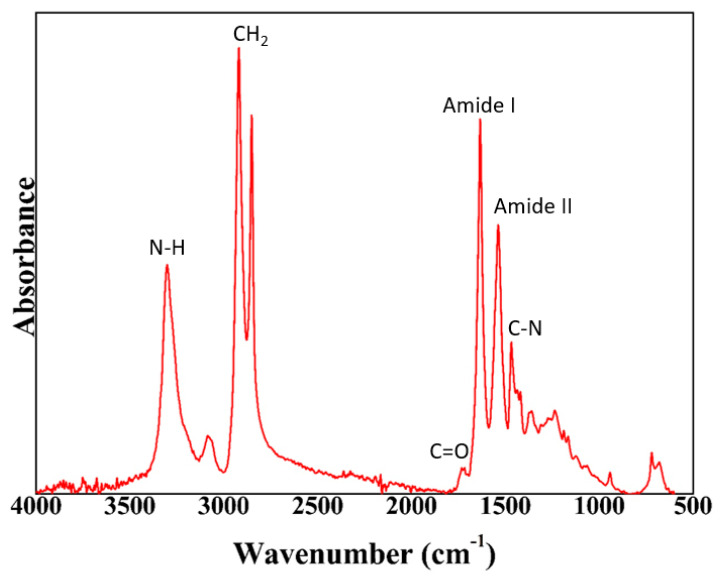
FTIR spectrum of the bio-based copolyamide PA1010/1014.

Figure 2 shows the ATR–FTIR spectrum of the produced bio-based copolyamide. The main characteristic peaks of polyamides were observed at 3303 cm^−1^ and 1633 cm^−1^, which are attributed to the amine (N–H) and the carbonyl (C=O) bands, respectively [27]. The latter is ascribed to Amide I in both the α- and β-crystalline phases, whereas the strong peak at 1537 cm^−1^ corresponds to the bending vibration of the N–H bond in Amide II. Furthermore, the band at 1471 cm^−1^ can be related to the contribution of the C–N stretching vibration of the amide groups. The peaks that were associated with the aliphatic chain, the bands at ca. 2849–2919 cm^−1^, were also very intense, which is in line with what was expected for the chemical structure of a polyamide with a relatively high methylene-to-amide (CH_2_/CONH) ratio. The resultant infrared spectrum was very similar to that which has been reported for the other bio-based polyamides, particularly polyamide 1010 (PA1010), but with a lower relative intensity of the bands that were ascribed to the crystalline forms [28]. This suggests a lower amide density with a potentially higher segmental inter-chain mobility, due to the more asymmetrical chain structure resulting from the presence of the comonomer in the polyamide chain structure.

The VN that was obtained for the synthesized biopolyamide was 67 cm^3^/g, which corresponds to an M_W_ in the range of 2.0–6.5·10^4^ g/mol, according to the Mark–Houwink parameters that have been reported for PA6 and PA12. This value is low compared to the commercial polyamides for extrusion and injection molding applications; however, in contrast, this can facilitate its use as tie or inner layers in multilayer structures, due its lower sealing initiation temperature (SIT). In this regard, the DSC results (not shown) showed that the obtained copolyamide had a melting point of 167.32 °C and a melting enthalpy of 45 J/g, yielding a crystallinity percentage (X_c_) of nearly 18%. This value of crystallinity is much lower than that which has been reported for PA1010, i.e., 27% [28], which is one of its corresponding homopolyamides, and even more than the short-chain aliphatic polyamides, such as polyamide 6 (PA6), i.e., 32–35% [29]. Therefore, the copolyamide that was obtained by this process corresponds to a long-chain aliphatic type, with a significantly lower melting point than the other conventional polyamides, such as PA6, which melts near 220 °C. A copolyamide with a similar melting profile was recently developed by Baniasadi et al. [30] by the polycondensation reaction of Sba and 1,12-diaminododecane with 11-aminoundecanoic acid to form PA11/1210 with a Tm of 158 °C. The copolyamide that has been developed herein presents a melting point that is considerably lower than that reported for PA6/66, with values still slightly above 200 °C [31]. In food packaging applications, the low melting point of the PA1010/1014 allows the combination of this semi-crystalline gas barrier polymer with biopolymers such as PHBV, which was used in this study (with a melting point of 174 °C [32]).

### 3.2. Solution Characterization

Surface tension, conductivity, and viscosity are the main solution parameters that affect the electrospinning process. The characterization of the prepared PHBV, the PA1010/1014 solutions, and the SiO_2_ dispersion in terms of these parameters is shown in Table 2. The neat PHBV solution showed the highest viscosity value, with a value of nearly 2800 cP, which is in accordance with a previous study that was conducted by Melendez-Rodriguez et al. [33]. In this case, the high viscosity could be related to the high M_W_ of the PHBV, according to Palangetic et al. [34], who studied the effect of polymer M_W_ on solution viscosity and its influence on the electrospinning process. Both the PA1010/1014 solution and the SiO_2_ dispersion showed significantly lower viscosities in comparison to PHBV. With regard to the surface tension, all of the solutions and the dispersion were below 50 mN/m; therefore, they were suitable for a stable electrospinning process [35]. Finally, all of them showed similar low conductivity values, ranging from 1 to 2 µS/cm.

### 3.3. Morphology of Electrospun Mono- and Multilayer Structures

**Figure 3:**
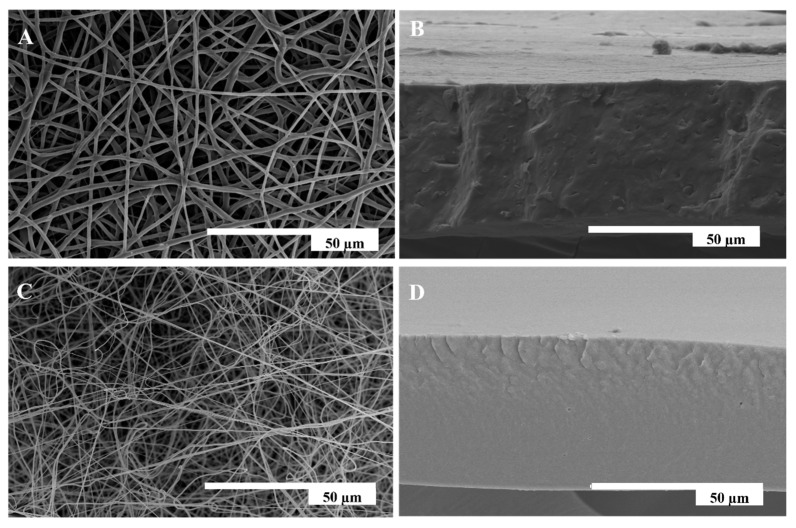
SEM images of the fibers and cross-section of the resulting films after annealing for (**A***)* fibers of PHBV; (**B**) annealed PHBV film at 125 °C; (**C**) fibers of PA1010/1014; and (**D**) annealed PA1010/1014 film at 120 °C.

First, an optimization of the electrospinning parameters was made in order to produce dry and straight fibers without the presence of beads. Figure 3 shows the morphologies of the electrospun and electrosprayed coatings that were studied by SEM, which were obtained with the optimized parameters that are shown in Table 3. A full characterization of the morphology, the thermal properties, and the particle size of the organomodified SiO_2_ particles has been reported previously [12]. Figure 3A,C show the top view of both the electrospun PHBV and the PA10101/1014 fiber mats, respectively. The microscopy results revealed the formation of smooth, bead-free, ultrathin fibers. The mean diameter of the PHBV fibers was 1.47 ± 0.49 µm. In the work of Figueroa-Lopez et al., the electrospun PHBV fibers were produced by dissolving 10% of the weight of the PHBV in TFE, the same conditions used in this work, obtaining a mean fiber diameter of approximately 0.95 µm [36]. For the biopolyamide, a mean fiber diameter of 0.83 ± 0.31 µm was obtained. Li et al. produced nylon 6,66,1010 electrospun fibers using similar conditions to those that have been used in this work, and they obtained an average fiber diameter of 0.38 µm [37]. The larger fiber diameter of PHBV can be related to the higher viscosity of the solution, as reported in Table 2. Figure 3B,D show the PHBV and PA1010/1014 fiber samples after the annealing treatment, respectively. This thermal post-treatment was performed at the minimum temperature, which was below the polymer melting point, that led to a continuous film [38,39]. Thus, the annealed samples showed a continuous structure that was produced by a thermally induced reduction in surface energy, triggering interfiber coalescence. In Figure 3B, it can be seen that the microstructure revealed the presence of some platelets sticking out from the cryofracture surface, or pulled out from it, which is known to arise from the presence of the nucleating agent boron nitride. In Figure 3D, it can be seen that the PA1010/1014 monolayer exhibited a very smooth and continuous morphology, but with some small pores in the cross-section, suggesting that the annealing process for this material was not completely efficient in eliminating the porosity of the material.

**Figure 4:**
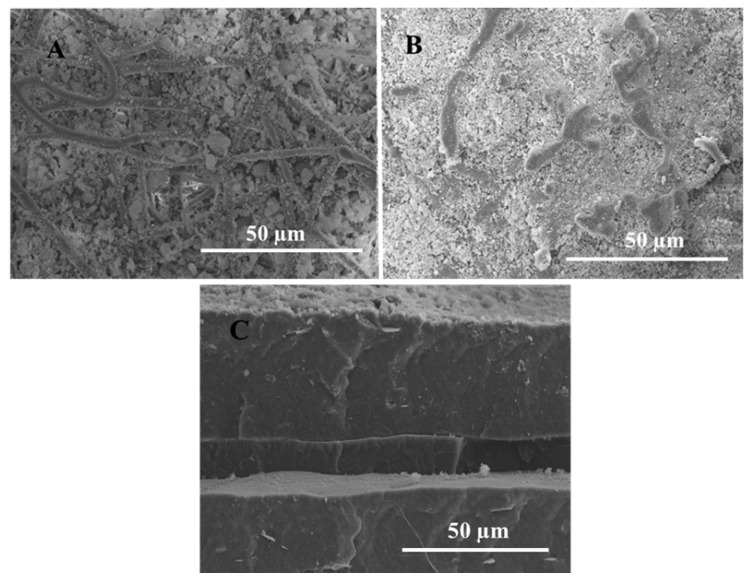
SEM images of: (**A**) the top view of SiO_2_ deposited on top of PHBV; (**B**) top view of SiO_2_ deposited on top of PHBV after annealing at 130 °C; and (**C**) cross-section of the multilayer film after annealing at 130 °C.

In Figure 4, the SEM morphology of the multilayer structure can be observed. Figure 4A shows the PHBV fibers that were coated with SiO_2_ particles, while Figure 4B shows the same sample after lamination. Finally, Figure 4C, shows the cross-section of the multilayer structure. In the cross-section image, it is possible to differentiate the three layers that form this multilayer film, each one with a continuous structure, suggesting the macroscopically observed good adhesion between the layers, despite the fact that an aggressive cryofracture was carried out before the SEM observation. A similar methodology was previously reported in the application of a protein inner layer using electrospinning, yielding high-barrier multilayer structures based on biopolymers [40].

### 3.4. Optical Properties

**Figure 5:**
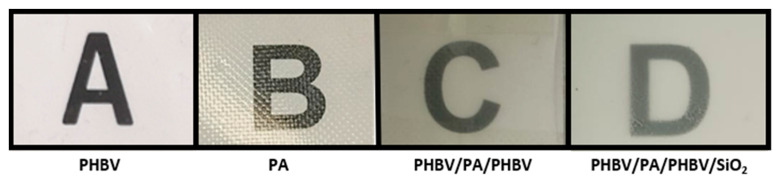
Visual aspect of the electrospun films of: (**A**) PHBV monolayer; (**B**) PA1010/1014 monolayer; (**C**) PHBV/PA1010/1014/PHBV multilayer; and (**D**) PHBV/PA1010/1014/PHBV/SiO_2_ multilayer.

The optical properties of the film are of paramount importance for the consumer to observe the food product through the packaging material. Figure 5 shows pictures of the aspect of the electrospun films in order to qualitatively characterize the samples according to their background transparency and Table 4 gathers the actual transparency values for the mono- and multilayer structures that were calculated using two methods. Regarding the transparency quantitative values, the monolayers showed better properties, with the PA1010/1014 film being more transparent, having the highest T value according to Equation (5), compared to that of PHBV. According to Equation (4), which takes the sample thickness into account, the PA1010/1014 film also showed an improved transparency, having the lowest T value according to Equation (4), but with no statistically significant difference compared to the PHBV film. A reduced transparency was seen for the case of the multilayers, with this effect being more pronounced in the presence of the SiO_2_ particles, which is probably due to the particles scattering some light [41]. However, the latter observation may also present some benefits for some food packaging materials, with the avoidance of oxidative reactions of lipids, carbohydrates, and proteins due to UV-light penetration.

### 3.5. Water Contact Angle and Sliding Angle of Mono- and Multilayer Structures

**Figure 6:**
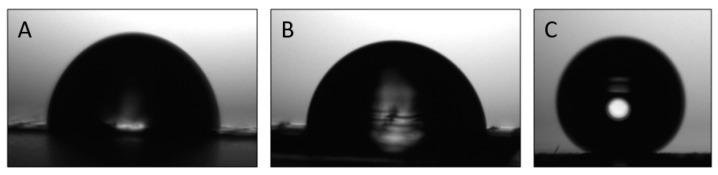
Typical photographs of the water contact angle test: (**A**) PHBV monolayer; (**B**) PA1010/1014 monolayer; and (**C**) PHBV/PA1010/1014/PHBV/SiO_2_ multilayer.

Table 5 gathers the results of the WCA. Figure 6 shows pictures of the contact angle test for each sample. Both of the polymer monolayer films that were made with PHBV or PA1010/1014 provided similar values in terms of the WCA. However, the incorporation of the silica nanostructured microparticles in the PHBV/PA1010/1014/PHBV/SiO_2_ multilayer film resulted in an apparent water contact angle value of 146.2° ± 0.2° and a sliding angle of 8°, results that very closely approached a superhydrophobic material, which is typically considered when the WCA is higher than 150° and the sliding angle is smaller than 10 [23,24]. This result is in agreement with the results that were reported in a previous study, in which the production of a different bilayer coating containing electrosprayed nanostructured SiO_2_ microparticles was studied [8]. The deposition of SiO_2_ generated a hierarchical roughness on the surface of the multilayer structure, allowing the values to reach close to the values that are characteristic of a superhydrophobic coatings after lamination. The resultant highly repellent surfaces can play an important role in many applications, such as contamination prevention, biocompatibility, easy emptying, enhanced lubricity, reduced food scalping, and the durability of the materials [42].

### 3.6. Mechanical Properties

**Figure 7:**
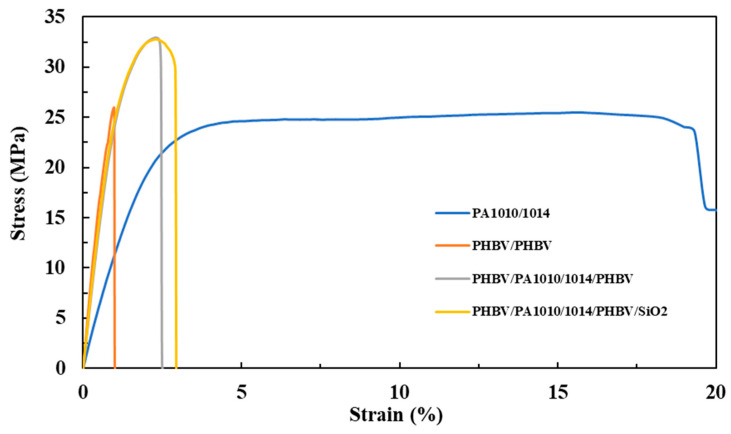
Typical tensile stress–strain curves of the annealed electrospun films of: PHBV bilayer; PA1010/1014 monolayer; PHBV/PA1010/1014/PHBV multilayer; and PHBV/PA1010/1014/PHBV/SiO_2_ multilayer.

The mechanical properties of the prepared films were characterized in terms of the tensile modulus (E), the tensile strength at yield (σ_y_), and the elongation at break ε_b_ (%). The obtained stress–strain curves are shown in Figure 7. All of the obtained values are summarized in Table 6. The values that were obtained for the PHBV/PHBV film are in concordance with those that have been reported previously by Melendez-Rodriguez et al. [43]. Regarding the electrospun PA1010/1014 film, in comparison with the PHBV film, it showed a lower tensile modulus and a slightly higher tensile strength at yield. In regard to the elongation at break, the value that was obtained was considerably greater. This result indicates that PA1010/1014 yields to the formation of a ductile but still resistance film. This behavior is in accordance with the work of Feng et al. [44], who found that, by increasing the 1014 unit content in a PA10T, an outstanding elongation at break could be observed. Therefore, the presence of the 1014 aliphatic units in the polyamide structure allows the improvement of the ductility of the material by reducing the crystallinity and amide bond density. Regarding the multilayer structure, these film samples showed intermediate modulus values in comparison to their corresponding neat monolayers; however, they were closer to those of the dominant PHA layer in the multilayer. Regarding the tensile strength at yield, the multilayer structure showed increased values in comparison to the neat biopolymer monolayer films. This phenomenon may be related to the presence of the copolyamide and a good interlayer adhesion in the multilayers, which may help to increase the elastic deformation range of the multilayers in comparison with the neat PHA, which shows a high modulus but also fragility. Furthermore, non-significant differences were observed in the mechanical performance of the multilayer after the incorporation of the SiO_2_, as expected for a thin coating. In conclusion, the multilayers showed a more ductile, and somewhat less mechanically resistant, structure than their corresponding neat PHBV monolayer film, mainly due to the presence of the PA1010/1014 layer.

### 3.7. Barrier Properties

The barrier performance is one of the most important parameters of application interest in food packaging [45]. The measured permeance values to water vapor and oxygen for the mono- and multilayer samples are shown in Table 7. The PHBV/PHBV bilayer film showed a good water vapor permeance result, which is in agreement with data that were reported earlier by Melendez-Rodriguez et al. [43]. Moreover, the literature reports a water permeability value of 1.7 × 10^−15^ kg m m^−2^ Pa^−1^ s^−1^ at 38 °C and 50% RH for the homopolymer PHB [46]. The annealed electrospun PHBV bilayer that has been produced here has a water vapor permeability of 1 × 10^−15^ kg m m^−2^ s^−1^ Pa^−1^. Regarding the PA1010/1014 film, it showed a lower barrier to water vapor in comparison to the PHBV/PHBV bilayer film. Thus, the water vapor permeability value at 23 °C and 100% RH for the annealed electrospun PA1010/1014 film that was synthesized in this work was 116 × 10^−15^ kg m m^−2^ s^−1^ Pa^−1^. The existing literature reports that PA6 has a water vapor permeability of 8.5 × 10^−15^ kg m m^−2^ s^−1^ Pa^−1^ when it was measured at 23 °C and 50% RH, while a synthesized long chain PA6,18 displayed a water vapor permeability of 2.3 × 10^−15^ kg m m^−2^ Pa^−1^ s^−1^ when it was measured at 23 °C and 50% RH [15]. The water vapor permeability at 38 °C and 90% RH for PA6 has been reported to be at 20.6 × 10^−15^ kg m m^−2^ s^−1^ Pa^−1^ [46]. From the comparative results, the annealed electrospun PA1010/1014 sample exhibited a lower water barrier than expected, most likely due to some porosity that was retained after the interfiber coalescence (see also the comparatively higher error of the measurements that have been provided for this material).

Interestingly, sandwiching the PA1010/1014 layer between two PHBV layers in the film structure reduced the water vapor permeance and also the error, which are results that could be related to a more compact layer morphology and confining between the water-resistant PHBV layers. The water permeability was, however, not reduced in the four-layer sample, suggesting that the silica-coated multilayer was not as efficient in blocking water vapor. Table 7 also shows the values of water uptake for the different samples. From this table, it can be observed that the PHBV bilayer presented an expected reduced water uptake in comparison to the neat PA1010/1014. The water sorption of the PHBV/PHBV film was in agreement with the value that was reported for PHBV by Li Wang et al. [47] of 0.26 %wt. The water uptake in polyamides is related to their chemical structure, since the amide linkages undergo hydrogen bonding, not only between the polymer chains, but also with sorbed water molecules. Sorbed moisture within polyamides acts as a plasticizing agent that reduces the interaction bonding and entanglement between the polymer chains. This results in an increase in “free” (unoccupied) volume and chain mobility [15], reducing the barrier properties. For instance, the water uptake result for the polyamide that has been synthesized in this work is in agreement with the result that was reported by Quiles-Carrillo et al. [28], who reported a value of 1.2 wt.-% for a PA1010 film. Other authors have reported water sorption values of 0.95 wt.-% for a PA1012 film [28], 0.80 wt.-% for a PA6,18 film [15], and 8.5 wt.-% for a PA6 film [48,49]. In the case of the three-layer and four-layer samples, a reduction in the water uptake was seen, with no significant difference among them.

Table 7 also presents the oxygen permeance data. The oxygen barrier result that was attained for the PHBV bilayer is in agreement with the work of Torres-Giner et al. [49], who reported a value of 0.80 × 10^−18^ m^3^ m m^−2^ s^−1^ Pa^−1^ for a compression-molded sheet of PHBV, with the value obtained in this work being in the same units of 0.72 × 10^−18^ m^3^ m m^−2^ s^−1^ Pa^−1^.

Regarding the PA1010/1014 film, similarly to the water vapor permeance result, an increase in oxygen permeance and in its corresponding error, in comparison with the PHBV bilayer, was measured. In this sample, the measurement was performed at 0% RH and at 25 °C because, at higher relative humidities, overrange measurements were given in the oxygen permeability tester. This suggests, again, that testing the annealed electrospun biopolyamide monolayer is not very reproducible, due to the above-mentioned remnant porosity. Making a comparison with the other polyamides that have been reported in the literature, PA6 shows oxygen permeability values of 0.05 × 10^−18^ m^3^ m m^−2^ s^−1^ Pa^−1^ at 0% RH [46] and 0.09 × 10^−18^ m^3^ m m^−2^ s^−1^ Pa^−1^ at 50% RH [15,50], and aliphatic long-chain polyamides show oxygen permeability values of 0.5 × 10^−18^ m^3^ m m^−2^ s^−1^ Pa^−1^ for a PA1010 film at 50 % RH [51] and 1.9 × 10^−18^ m^3^ m m^−2^ s^−1^ Pa^−1^ for a PA6,18 film at 50 % RH [15]. On the other hand, our annealed electrospun PA1010/1014 film yielded a value of 2.3 × 10^−18^ m^3^ m m^−2^ s^−1^ Pa^−1^, clearly higher than would be expected.

In the case of the three-layer sample, the introduction of the PA1010/1014 inner layer between the two PHBV layers reduced the oxygen permeance by 44% in a thicker sample. Therefore, the barrier effect is not brought by the electrospun biopolyamide being a better oxygen barrier than PHBV in this case, as was the case with the water vapor barrier, but by the thickness increase in the multilayer. The incorporation of the silica coating in the four-layer sample showed, as with the water barrier, lower permeability improvement, which is most likely due to a combination of a more heterogeneous packing morphology with higher diffusion and lower thickness for this multilayer.

## 4. Conclusions

This study has presented for the first time the formulation of electrospun multilayers of a fully bio-based aliphatic copolyamide 1010/1014 with a low melting point together with electrospun PHBV and electrosprayed food contact organomodified SiO_2_ nanostructure microparticles, which, after lamination, exhibited a good oxygen and water vapor barrier (5.16 × 10^−15^ m^3^ m^−2^ s^−1^ Pa^−1^ and 1.02 × 10^−11^ kg m^−2^ s^−1^ Pa^−1^, respectively), interlayer adhesion, improved mechanical properties compared to the neat PHBV, and close to superhydrophobic behavior, i.e., an apparent water contact angle of ca. 146° and a sliding angle of 8°. It was found that the annealed electrospun synthesized copolyamide could be laminated at 130 °C, a temperature that is compatible with the PHBV biopolyester that was selected; however, it did not show the expected gas barrier results as a monolayer, which was most likely due to inefficient interfiber coalescence resulting in remnant porosity. Overall, the multilayers that have been developed can be potentially considered in a new route to develop more sustainable strategies for packaging applications.

## Figures and Tables

**Table 1:** Thermal post-treatment conditions for the mono- and multilayer films.

Sample	Temperature (°C)	Roll Speed (m/min)
PA1010/1014	120	0.52
PHBV	125	0.52
PHBV/PA1010/1014/PHBV	130	0.52
PHBV/PA1010/1014/PHBV/SiO_2_	130	0.52

**Table 2:** Properties of the PHBV, PA1010/1014 solution, and SiO_2_ dispersion.

Sample	Surface Tension (mN/m)	Conductivity (µS/cm)	Viscosity (cP)
PHBV	31.0 ± 3.8 ^a^	1.79 ± 0.03 ^a^	2799.8 ± 45.7 ^a^
PA1010/1014	19.4 ± 0.6 ^b^	1.29 ± 0.07 ^a^	640.3 ± 4.2 ^b^
SiO_2_	26.3 ± 1.3 ^a^	1.33 ± 0.99 ^a^	339.2 ± 8.3 ^c^

^a–c^ Different letters in the same column indicate a significant difference among the samples (*p <* 0.05).

**Table 3:** Electrospinning parameters for PHBV and PA1010/1014 and the electrospraying of SiO_2_ microparticles.

Processing Parameters	PHBV Solution	BioPA Solution	SiO_2_ Dispersion
Flowrate per needle (mL/h)	4.0	2.4	1.2
Voltage emitter (kV)	10.0	15.0	5.0
Voltage collector (kV)	22.5	20.0	18.0
Distance collector (cm)	20.0	23.5	22.5
Drum speed (rpm)	200	200	200
Temperature (°C)	30	30	30
Relative humidity (%)	30	30	30

**Table 4:** Transparency data of the mono- and multilayer films of PHBV, PA1010/1014, and SiO_2_ microparticles.

Sample	T (%) (Equation (5))	T (mm^−1^) (Equation (4))
PHBV	23.7 ± 1.4 ^a^	9.9 ± 2.3 ^a^
PA1010/1014	52.1 ± 0.3 ^b^	8.1 ± 0.9 ^a^
PHBV/PA1010/1014/PHBV PHBV/ PA1010/1014/PHBV/SiO_2_	6.5 ± 1.9 ^c^ 2.6 ± 0.1 ^d^	10.8 ± 0.3 ^a,b^ 14.0 ± 0.1 ^b^

^a–d^ Different letters in the same column indicate a significant difference among the samples (*p <* 0.05).

**Table 5:** WCA values of the mono- and multilayer films of PHBV, PA1010/1014, and SiO_2_ microparticles.

Sample	WCA (°)
PHBV	82.5 ± 1.0 ^a^
PA1010/1014	86.3 ± 0.2 ^b^
PHBV/PA1010/1014/PHBV/SiO_2_	146.2 ± 0.2 ^c^

^a–c^ Different letters in the same column indicate a significant difference among the samples (*p* < 0.05).

**Table 6:** Mechanical properties of the mono- and multilayer films based on PHBV, PA1010/1014, and SiO_2_.

Samples	E (MPa)	σ_y_ (MPa)	ε_b_ (%)
PHBV/PHBV	3347 ± 261 ^a^	22.0 ± 6.7 ^a^	1.0 ± 0.1 ^a^
PA1010/1014	1202 ± 246 ^b^	25.2 ± 3.1 ^a,b^	21.9 ± 4.4 ^b^
PHBV/ PA1010/1014/PHBV	2925 ± 86 ^a^	33.3 ± 2.4 ^b^	2.4 ± 0.4 ^a^
PHBV/ PA1010/1014/PHBV/SiO_2_	2898 ± 162 ^a^	32.3 ± 1.1 ^b^	2.8 ± 0.3 ^a^

^a–b^Different letters in the same column indicate a significant difference among the samples (*p <* 0.05).

**Table 7:** Mean thickness, water vapor and oxygen permeances, and water uptake of the mono- and multilayer films based on PHBV, PA1010/1014, and SiO_2_.

Sample	Thickness (µm)	WVP × 10^11^ (kg m^−2^s^−1^Pa^−1^)	OP × 10^15^ (m^3^ m^−2^s^−1^Pa^−1^)	Water Uptake (%wt)
PHBV/PHBV	97 ± 5 ^a^	1.04 ± 0.30 ^a^	7.00 ± 0.07 ^a^	0.20 ± 0.20 ^a^
PA1010/1014	72 ± 8 ^b^	161 ± 68 ^b^	31.3 ± 7.0 ^b,^*	1.10 ± 0.53 ^b^
PHBV/PA1010/1014/PHBV	184 ± 13 ^c^	0.30 ± 0.03 ^a^	3.95 ± 0.40 ^a,c^	0.57 ± 0.21 ^a,b^
PHBV/ PA1010/1014/PHBV/SiO_2_	160 ± 8 ^d^	1.02 ± 0.06 ^a^	5.16 ± 0.02 ^a,d^	0.38 ± 0.17 ^a,b^

^a–d^ Different letters in the same column indicate a significant difference among the samples (*p* < 0.05). * Sample measured at 0% RH, 25° C.
